# Triazole Evolution of Candida parapsilosis Results in Cross-Resistance to Other Antifungal Drugs, Influences Stress Responses, and Alters Virulence in an Antifungal Drug-Dependent Manner

**DOI:** 10.1128/mSphere.00821-20

**Published:** 2020-10-28

**Authors:** Csaba Papp, Flóra Bohner, Katica Kocsis, Mónika Varga, András Szekeres, László Bodai, Jesse R. Willis, Toni Gabaldón, Renáta Tóth, Joshua D. Nosanchuk, Csaba Vágvölgyi, Attila Gácser

**Affiliations:** a Department of Microbiology, Faculty of Science and Informatics, University of Szeged, Szeged, Hungary; b Department of Biochemistry and Molecular Biology, Faculty of Science and Informatics, University of Szeged, Szeged, Hungary; c Barcelona Supercomputing Centre (BSC-CNS), Jordi Girona, Barcelona, Spain; d Institute for Research in Biomedicine (IRB Barcelona), The Barcelona Institute of Science and Technology, Baldiri Reixac, Barcelona, Spain; e Catalan Institution for Research and Advanced Studies (ICREA), Barcelona, Spain; f Division of Infectious Diseases, Department of Medicine, Albert Einstein College of Medicine, Bronx, New York, USA; g Department of Microbiology and Immunology, Albert Einstein College of Medicine, Bronx, New York, USA; h MTA-SZTE Lendület Mycobiome Research Group, University of Szeged, Szeged, Hungary; University of Georgia

**Keywords:** *Candida*, antifungal resistance, triazole, virulence

## Abstract

The number of invasive infections caused by *Candida* species is increasing worldwide. The incidence of candidiasis cases caused by non-*albicans Candida* species, such as Candida parapsilosis, is also increasing, and non-*albicans Candida* species are currently responsible for more invasive infections than C. albicans. Additionally, while the development of azole resistance during invasive disease with C. albicans remains uncommon, azole-resistant C. parapsilosis strains are frequently isolated in the hospital setting. In this study, we applied direct selection to generate azole-adapted and azole-evolved C. parapsilosis strains in order to examine the effect of azole resistance development on fungal viability and pathogenesis progression. Depending on the drug applied, the different evolved strains developed distinct cross-resistance patterns: the fluconazole-evolved (FLU^EVO^) and voriconazole-evolved (VOR^EVO^) strains gained resistance to fluconazole and voriconazole only, while posaconazole evolution resulted in cross-resistance to all azoles and the posaconazole-evolved (POS^EVO^) strains showed higher echinocandin MIC values than the FLU^EVO^ and VOR^EVO^ strains. Whole-genome sequencing results identified the development of different resistance mechanisms in the evolved strains: the FLU^EVO^ and VOR^EVO^ strains harbored amino acid substitutions in Mrr1p (A808T and N394Y, respectively), and the POS^EVO^ strain harbored an amino acid change in Erg3p (D14Y). By revealing increased efflux pump activity in both the FLU^EVO^ and the VOR^EVO^ strains, along with the altered sterol composition of the POS^EVO^ strain, we now highlight the impact of the above-mentioned amino acid changes in C. parapsilosis azole resistance development. We further revealed that the virulence of this species was only slightly or partially affected by fluconazole and voriconazole adaptation, while it significantly decreased after posaconazole adaptation. Our results suggest that triazole adaptation can result in azole cross-resistance and that this process may also result in virulence alterations in C. parapsilosis, depending on the applied drug.

**IMPORTANCE** Candida parapsilosis causes life-threatening fungal infections. In the last 2 decades, the increasing number of azole-resistant C. parapsilosis clinical isolates has been attributable to the overuse and misuse of fluconazole, the first-line antifungal agent most commonly used in several countries. To date, the range of applicable antifungal drugs is limited. As a consequence, it is essential to understand the possible mechanisms of antifungal resistance development and their effect on virulence in order to optimize antifungal treatment strategies in the clinical setting. Our results revealed that the prolonged exposure to azoles resulted not only in azole resistance but also in cross-resistance development. Our data further indicate that resistance development may occur through different mechanisms that can also alter the virulence of C. parapsilosis. These results highlight the consequences of prolonged drug usage and suggest the need for developing alternative antifungal treatment strategies in clinical practice.

## Abstract (precis)

Candida parapsilosis causes life-threatening fungal infections. In the last 2 decades, the increasing number of azole-resistant C. parapsilosis clinical isolates has been attributable to the overuse and misuse of fluconazole, the first-line antifungal agent most commonly used in several countries. To date, the range of applicable antifungal drugs is limited. As a consequence, it is essential to understand the possible mechanisms of antifungal resistance development and their effect on virulence in order to optimize antifungal treatment strategies in the clinical setting. Our results revealed that the prolonged exposure to azoles resulted not only in azole resistance but also in cross-resistance development. Our data further indicate that resistance development may occur through different mechanisms that can also alter the virulence of C. parapsilosis. These results highlight the consequences of prolonged drug usage and suggest the need for developing alternative antifungal treatment strategies in clinical practice.

## INTRODUCTION

The number of invasive fungal infections is increasing worldwide (1), and *Candida* species are the most common mycological cause of bloodstream infections (2). Candida albicans remains the leading cause of invasive disease; however, the number of infections caused by other *Candida* species, such as C. parapsilosis, C. glabrata, C. tropicalis, and C. krusei, has increased markedly in the last 3 decades (2–5). Currently, echinocandins are recommended as the first-line antifungal agents for the treatment of systemic candidiasis (6–8), although triazoles, especially fluconazole, are commonly utilized when the infecting species is susceptible to these agents. Although the majority of C. parapsilosis clinical isolates are susceptible to azole antifungals, they have the highest *in vitro* MIC values for echinocandins compared to other *Candida* species (7). Despite the concern over high echinocandin MIC values *in vitro*, patients with C. parapsilosis infection generally respond well to echinocandin treatment (9). In a previous study, we revealed how this paradoxical phenomenon can be explained: acquired echinocandin resistance leads to attenuated virulence *in vivo*, possibly due to a fitness cost (10). Interestingly, while C. albicans clinical isolates are rarely resistant to azole derivatives, azole-resistant C. parapsilosis and C. glabrata strains are frequently isolated from patients (11). Additionally, the rate of azole resistance among C. parapsilosis isolates has increased over the past 2 decades (11–13). Prior to the introduction of echinocandins in general practice, fluconazole was the first-line antifungal agent for candidiasis treatment worldwide and is still prophylactically administered to certain patients (14, 15). Azole cross-resistance (mostly between fluconazole and voriconazole) occurs in approximately two-thirds of fluconazole-resistant C. parapsilosis isolates (16, 17).

Despite the emergence of azole-resistant C. parapsilosis clinical isolates, the molecular mechanisms of azole resistance have been investigated mainly in C. albicans (a species not typically associated with azole resistance during invasive disease) and in C. glabrata, while only a limited number of studies have focused on C. parapsilosis (1, 18, 19). The development of resistance in *Candida* species has been described to be a result of mutations in or the overexpression of ergosterol biosynthetic enzymes (e.g., Erg11) or due to the overexpression of efflux pump-encoding genes (*MDR1* and *CDR1*) as a consequence of mutations in their corresponding transcription regulatory factors (*MRR1* and *TAC1*, respectively) (19, 20). However, species-specific investigations are required, as certain mechanisms are not globally effective across the genus. In C. albicans, the Upc2 transcription factor is responsible for *ERG11* overexpression. In contrast, this transcription factor does not possess the same role in C. parapsilosis, but, rather, it is required for the overexpression of other ergosterol biosynthesis genes (19, 20). Similarly, previous studies have shown that while *MDR1* and *CDR1* overexpression is important in terms of acquiring azole resistance in C. albicans, *MDR1* and *CDR1* play a minor role in resistance development in C. parapsilosis (18, 21). Based on this information, we aimed to investigate the specific adaptation mechanisms of C. parapsilosis in response to long-term exposure to triazoles and the impact of the acquired resistance on virulence *in vivo*.

## RESULTS

### Azole-evolved C. parapsilosis strains developed altered susceptibility to different antifungals, depending on the drug used for microevolution.

**FIG 1:**
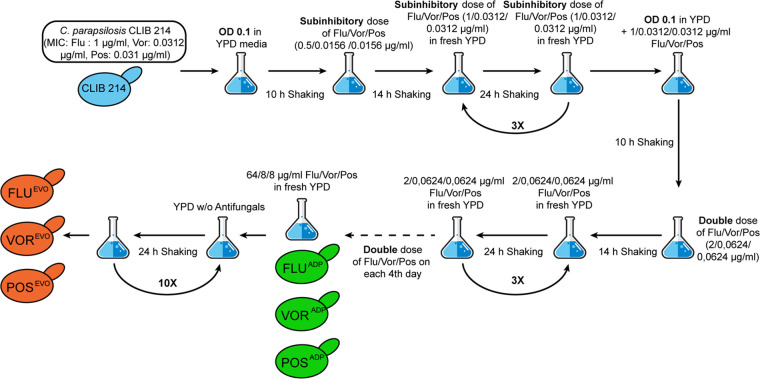
Process for the generation of triazole-adapted (ADP) and -evolved (EVO) strains.

Prior to the microevolution process, the MIC values of fluconazole (FLU; 1 μg/ml), voriconazole (VOR; 0.031 μg/ml), and posaconazole (POS; 0.031 μg/ml) for the C. parapsilosis CLIB 214 strain were determined (Table 1). Next, we generated the adapted (ADP) strains by incubating CLIB 214 cells in the presence of increasing amounts of one of the three different antifungal drugs. Following direct selection, the evolved (EVO) strains were derived from the adapted strains by repeated culturing in yeast extract-peptone-dextrose (YPD) without the previously used antifungal following a scheme that we previously described (10) and that is shown in Fig. 1.

**TABLE 1:** MIC values of Candida parapsilosis parental, adapted, and evolved strains

Strain	MIC (μg/ml)															
	AMP		FLU		VOR		POS		ITR		CAS		AND		MICA	
	24 h	48 h	24 h	48 h	24 h	48 h	24 h	48 h	24 h	48 h	24 h	48 h	24 h	48 h	24 h	48 h
CLIB 214	0.5	0.25	1	1	0.031	0.031	0.031	0.031	0.063	0.063	1	1	1	2	1	2
FLU^ADP^	1	1	128	128	2	2	0.031	0.031	0.125	0.25	1	1	1	1	0.5	2
FLU^EVO^	1	2	128	128	2	2	0.031	0.031	0.25	0.25	1	1	1	1	0.5	2
VOR^ADP^	0.5	0.5	>256	>256	8	8	0.031	0.031	0.25	0.25	0.5	0.5	1	1	0.5	1
VOR^EVO^	1	2	>256	>256	8	8	0.031	0.031	0.25	0.25	1	1	1	1	0.5	1
POS^ADP^	0.5	1	>256	>256	>32	>32	1	2	4	4	2	2	2	2	4	4
POS^EVO^	1	1	>256	>256	>32	>32	>32	>32	>32	>32	2	4	4	4	4	8

**FIG 2:**
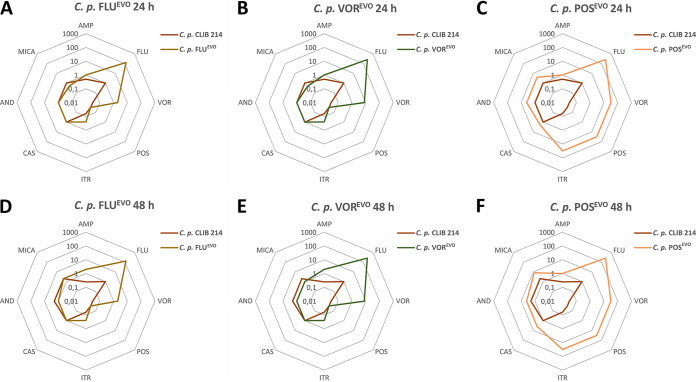
Spider web diagram representation of the changes in the antifungal susceptibilities of the C. parapsilosis (*C. p.*) FLU^EVO^ (A, D), VOR^EVO^ (B, E), and POS^EVO^ (C, F) strains after 24 h (A to C) and 48 h (D to F).

The susceptibilities of the generated triazole-adapted and -evolved strains to amphotericin B (AMP), azoles (FLU, VOR, POS, itraconazole [ITR]), and echinocandins (caspofungin [CAS], anidulafungin [AND], micafungin [MICA]) were tested. Adapted (ADP) strains were defined as cell populations grown in the presence of the maximal concentration of a specific azole used during the microevolution process. Evolved (EVO) strains were derived from ADP strains following their serial cultivation 10 times in YPD without antifungals. Most of the adapted strains showed MIC values similar to those for their corresponding evolved strains, except for the POS^ADP^ and POS^EVO^ strains, as the MICs of POS and ITR were much higher for the POS^EVO^ strain than for the POS^ADP^ strain (Table 1). The MIC values of AMP for all adapted and evolved strains increased 2- to 3-fold compared to those for the parental C. parapsilosis strain. There were slight decreases in the echinocandin MIC values for the FLU^ADP^, FLU^EVO^, VOR^ADP^, and VOR^EVO^ strains (Fig. 2A, B, D, and E). However, the POS^ADP^ and POS^EVO^ strains showed 2- to 4-fold increases in the MICs of the echinocandins (Fig. 2C and F). All azole-ADP and -EVO strains developed resistance to FLU. Notably, the VOR^ADP^, VOR^EVO^, POS^ADP^, and POS^EVO^ strains all possessed higher MIC values (>256 μg/ml for fluconazole) than the FLU^ADP^ or FLU^EVO^ strains (Fig. 2A to F). All generated strains gained resistance to VOR, with increasing MICs being seen for the FLU^ADP^/FLU^EVO^ strains (2 μg/ml) and then the VOR^ADP^/VOR^EVO^ strains (8 μg/ml) and the POS^ADP^ and POS^EVO^ strains (>32 μg/ml), for which the values were the highest (Table 1; Fig. 2). The adapted and evolved strains generated using FLU or VOR did not display cross-resistance to POS. In the case of ITR, the FLU^ADP^, FLU^EVO^, VOR^ADP^, and VOR^EVO^ strains displayed 2-fold increases in MIC values (0.25 μg/ml), and the POS^ADP^ and POS^EVO^ strains developed resistance to this antifungal drug (MICs, 4 μg/ml and >32 μg/ml, respectively).

### Azole microevolution leads to altered stress tolerance.

We compared the abiotic stress tolerance of the evolved (EVO) strains to that of the CLIB 214 parental strain on solid agar plates with a spot assay. On YPD plates, no differences in growth between the EVO strains and the parental strain were detected (data not shown). In the presence of glycerol, NaCl, and sorbitol, we detected slight or moderate growth defects at 30°C for the VOR^EVO^ and POS^EVO^ strains. Attenuated growth was more pronounced at 37°C than at 30°C. Additionally, the FLU^EVO^ strain showed slight growth defects at 37°C in the presence of osmotic stressors.

**FIG 3:**
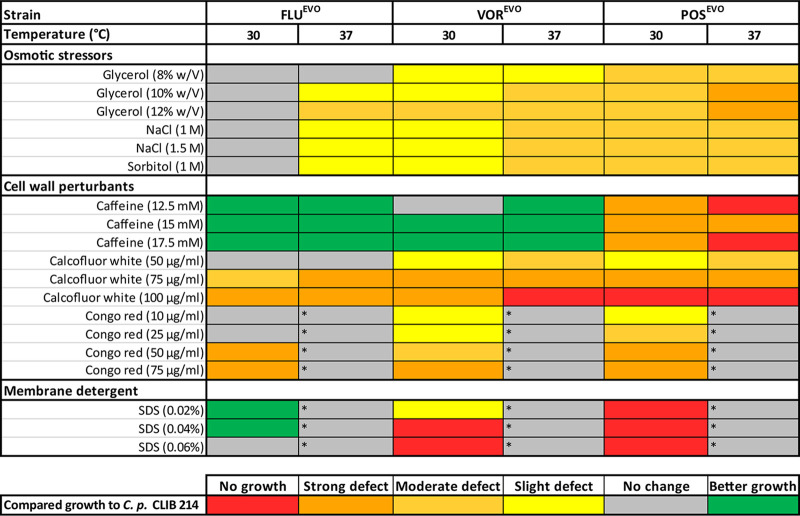
Growth of the triazole-evolved strains in the presence of abiotic stressors compared to the growth of the C. parapsilosis CLIB 214 parental strain. Asterisks indicate conditions under which the C. parapsilosis CLIB 214 parental strain was unable to grow.

Interestingly, the FLU^EVO^ and VOR^EVO^ strains were able to surpass the parental CLIB 214 strain in growth in the presence of caffeine at both 30 and 37°C. In contrast, the POS^EVO^ strain showed a strong growth defect or no growth at all on caffeine-containing plates. The growth of the VOR^EVO^ and POS^EVO^ strains slightly and moderately decreased, respectively, in the presence of 50-μg/ml calcofluor white (CW) at both 30°C and 37°C. CW at 75 μg/ml caused a strong growth defect in all triazole-evolved strains, and the growth defect was the most vigorous in the presence of 100-μg/ml CW in the case of VOR^EVO^ at 37°C and POS^EVO^ at both temperatures, where no growth was detectable. In the presence of Congo red (CR), none of the strains, including the parental C. parapsilosis CLIB 214 strain, were able to grow at 37°C (Fig. 3). Utilization of 10- and 25-μg/ml CR caused only a slight decrease in growth in the case of the VOR^EVO^ and POS^EVO^ strains; however, higher concentrations of CR (50 μg/ml, 75 μg/ml) resulted in a strong growth defect in all triazole-evolved strains.

Similarly, the membrane-perturbing compound sodium dodecyl sulfate (SDS) also caused the complete arrest of the growth of all strains at 37°C. Interestingly, the FLU^EVO^ strain showed improved growth compared to that of the CLIB 214 parental strain, while the VOR^EVO^ and POS^EVO^ strains were also unable to grow at 30°C (Fig. 3).

### Whole-genome sequencing (WGS) reveals different potential resistance mechanisms in the triazole-evolved strains.

To ascertain a potential mechanism of triazole resistance, we compared the genomes of the FLU^EVO^, VOR^EVO^, and POS^EVO^ strains to the genome of the parental strain. All point mutations are listed in Data Set S1 in the supplemental material. We detected 136, 144, and 142 nonsynonymous mutations in 121, 127, and 128 open reading frames (ORFs) in the genomes of the FLU^EVO^, VOR^EVO^, and POS^EVO^ strains, respectively, when their sequences were compared to the sequence of the parental C. parapsilosis CLIB 214 strain. Among these, 101 genes harbored at least 1 single nucleotide polymorphism (SNP) in all of the evolved strains. Inspection of the functional annotation of the genes harboring the highest values of SNPs per kilobase pair in all the experiments did not reveal any clear candidate related to the acquisition of resistance. Therefore, we focused on genes that harbored mutations in both the FLU^EVO^ and VOR^EVO^ strains simultaneously or genes that were mutated only in the POS^EVO^ strain and that according to their function may be directly responsible for the acquisition of resistance. The mutations that met these criteria are highlighted in Table 2. The FLU^EVO^ and VOR^EVO^ strains harbored point mutations in the CPAR2_807270 region (C. albicans orthologue, *MRR1*, the transcriptional activator of *MDR1*) at contig positions 1679479 and 1678237, causing the A808T and N394Y amino acid changes, respectively. These two strains also had the same nonsynonymous mutation in the CPAR2_100280 gene at contig position 45253, resulting in the F4L amino acid substitution. The POS^EVO^ strain harbored an SNP in the gene CPAR2_105550 potentially responsible for azole and echinocandin resistance. These SNPs occurred at contig position 1207930, leading to the D14Y amino acid change. CPAR2_105550 is a putative gene orthologous to C. albicans *ERG3*, which participates in ergosterol biosynthesis and which encodes a C-5 sterol desaturase.

**TABLE 2:** Amino acid substitutions in genes potentially responsible for resistance in C. parapsilosis triazole-evolved strains (FLU^EVO^, VOR^EVO^, POS^EVO^) compared to the parental CLIB 214 strain

Gene	Strain(s)	C. albicans orthologue	Orthologue function	Amino acid substitution
CPAR2_807270	FLU^EVO^, VOR^EVO^	*MRR1*	Activator of Mdr1 efflux pump	A808T (for FLU^EVO^), N394Y (for VOR^EVO^)
CPAR2_100280	FLU^EVO^, VOR^EVO^	C5_03940C_A	Putative multidrug resistance protein, upregulated by Efg1p	F4L
CPAR2_105550	POS^EVO^	*ERG3*	C-5 sterol desaturase, role in ergosterol biosynthesis	D14Y

10.1128/mSphere.00821-20.1 DATA SET S1 All identified nonsynonymous SNPs detected in the triazole-evolved strains. The genome of the parental C. parapsilosis CLIB 214 strain was used as a reference. Columns in the table indicate the following: #Coord, coordinates, indicating the contig number and position within contig of the given SNP; Ref, reference, indicating the allele in the reference strain; Genomic_region, exonic or intergenic; Gene, gene name when available; AA_change, amino acid change, when applicable (in the format *XYZ*, where *X* is the reference amino acid, *Y* is the position within the translated protein sequence, and *Z* is the alternative amino acid); [sample_name], when an SNP is present (in the format *a*, *b*, *c*, *d*, where *a* is the alternate allele, *b* is the zygosity, *c* is the coverage at the position, and *d* is the SNP class [when applicable]); and function, annotated gene function, when applicable. The first, second, and third data sheets contain the SNPs in the FLU^EVO^, VOR^EVO^, and POS^EVO^ strains, respectively. Download Data Set S1, XLS file, 0.3 MB. Copyright © 2020 Papp et al. 2020 Papp et al. This content is distributed under the terms of the Creative Commons Attribution 4.0 International license.

### The FLU^EVO^ and VOR^EVO^ strains show elevated efflux pump activity.

**FIG 4:**
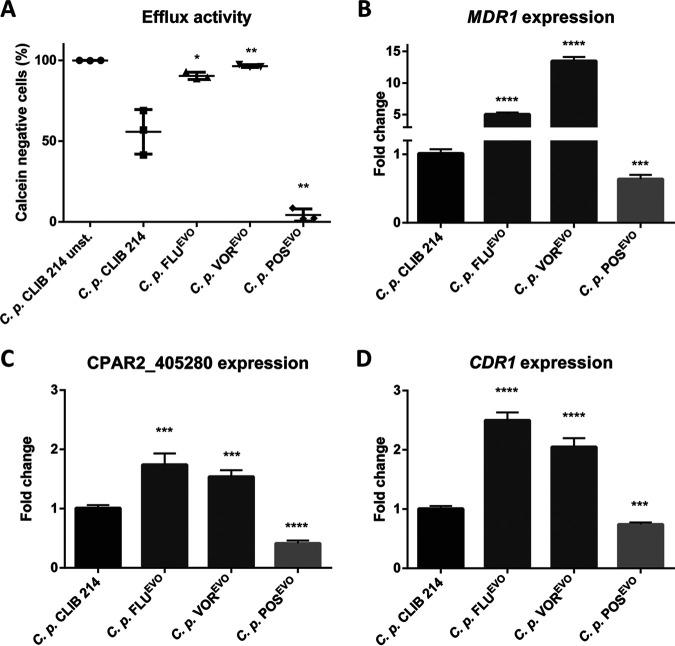
Efflux activity of C. parapsilosis CLIB 214, FLU^EVO^, VOR^EVO^, and POS^EVO^ strains. (A) Efficiency of calcein efflux from cells of the C. parapsilosis CLIB 214, FLU^EVO^, VOR^EVO^, and POS^EVO^ strains. (B to D) Normalized expression profile of *MDR1* (B), CPAR2_405280 (C), and *CDR1* (D). *, *P* ≤ 0.05; **, *P* ≤ 0.01; ***, *P* ≤ 0.001; ****, *P* ≤ 0.0001.

We next examined the efflux pump activity and the *MDR1* expression of the C. parapsilosis azole-evolved strains. Staining with the acetoxymethyl (AM) ester derivative of calcein (calcein-AM) is regularly used to determine efflux pump activity in yeasts (22). During calcein-AM staining, the intracellular esterases cleave the AM group, which leads to the inability of calcein to permeate the membrane, which results in its accumulation within a cell, producing a strong intracellular fluorescent signal. If the efflux pump activity is elevated within the host cell due to, e.g., the upregulation of the Mdr1 and Cdr1 proteins, calcein is transported into the extracellular space, and consequently, the intracellular fluorescent signal weakens. We found that the fluorescent signal decreased in the FLU^EVO^ and VOR^EVO^ strains compared to that in the parental CLIB 214 C. parapsilosis strain, suggesting an increase in efflux activity in these strains, which could be responsible for FLU and VOR cross-resistance development (Fig. 4A). Interestingly, efflux pump activity decreased in the POS^EVO^ strain compared to the parental strain (Fig. 4A). To support the findings of the calcein-AM staining, we compared the expression of *MDR1*, CPAR2_405280, and *CDR1* (CPAR2_405290) in the FLU^EVO^, VOR^EVO^, and POS^EVO^ strains to that in the parental C. parapsilosis strain. The expression levels in the azole-evolved strains were normalized to the expression levels measured in the C. parapsilosis CLIB 214 parental strain. We found that all three genes examined (CPAR2_405280, *CDR1*, and *MDR1*) were expressed at a higher level in the FLU^EVO^ and VOR^EVO^ strains than in the C. parapsilosis CLIB 214 strain. In accordance with the calcein-AM staining results, these genes were downregulated in the POS^EVO^ strain. We observed the highest *MDR1* expression levels in the FLU^EVO^ and VOR^EVO^ strains (5- and 13-fold increases, respectively), while *MDR1* expression was 1.58 times lower in the POS^EVO^ strain than in the C. parapsilosis CLIB 214 strain (Fig. 4B). CPAR2_405280 and *CDR1* expression also increased, although to a lower extent in the FLU^EVO^ and VOR^EVO^ strains than in the parental strain: 1.7- and 2.49-fold increases, respectively, were detected in the FLU^EVO^ strain than in the parental strain, and 1.54- and 2.05-fold increases, respectively, were observed in the VOR^EVO^ strain than in the parental strain. In contrast, CPAR2_405280 and *CDR1* expression levels decreased 2.44 and 1.36 times, respectively, in the POS^EVO^ strain compared to the C. parapsilosis CLIB 214 strain (Fig. 4C and D).

### Posaconazole evolution leads to an altered sterol composition possibly due to an *ERG3* loss-of-function mutation.

**FIG 5:**
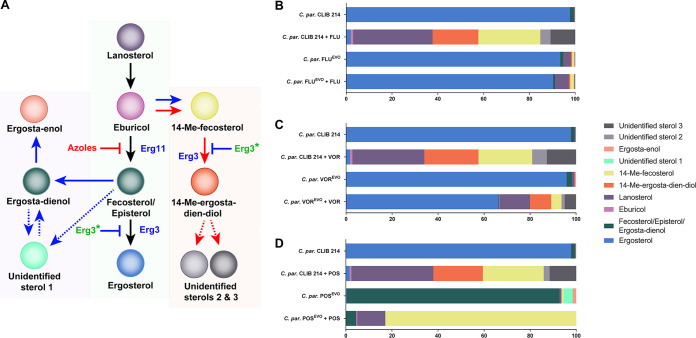
Altered sterol composition of the C. parapsilosis (*C. par*) CLIB 214, FLU^EVO^, VOR^EVO^, and POS^EVO^ strains in the presence and absence of fluconazole, voriconazole, or posaconazole. (A) Schematic of ergosterol biosynthesis (black arrows) and its alterations due to azole treatment (red arrows) and *ERG3* loss-of-function mutations (blue arrows). Dashed lines represent the unclear origin of recently detected, yet unidentified sterols. (B to D) Sterol composition of fluconazole-related (B), voriconazole-related (C), and posaconazole-related (D) samples.

For the validation of an *ERG3* loss-of-function mutation, we examined the sterol content of C. parapsilosis CLIB 214 parental and azole-evolved strains in the presence and absence of the appropriate azole. The relative sterol composition of each strain was determined by liquid chromatography–high-resolution mass spectrometry (LC-HRMS) and gas chromatography-mass spectrometry, and the raw data are available in Data Set S2. Figure 5A depicts a schematic of the ergosterol biosynthesis pathway, including the main detectable components derived from our sterol composition results. In the untreated parental C. parapsilosis CLIB 214 strain, the main sterol component was ergosterol (97.74%), while treatment with each of the azoles reduced the ergosterol content to below 2%. Instead of ergosterol, the most abundant sterol compounds were lanosterol (31.28 to 35.56%), 14-methyl (Me)-fecosterol (23.36 to 27.04%), and 14-Me-ergosta-dien-diol (which is toxic; 20.02 to 23.54%). Additionally, we also identified two as yet uncharacterized sterol compounds (US): US2 (2.57 to 6.37%) and US3 (10.74 to 12.7%) (Fig. 5B to D; Data Set S2). The FLU^EVO^ strain produced ergosterol in the presence (90.39%) or absence (93.45%) of FLU (Fig. 5B; Data Set S2). The pattern of the sterol composition of C. parapsilosis VOR^EVO^ azole-untreated cells showed similarities to that of untreated CLIB 214 cells and both treated and untreated FLU^EVO^ strains, as ergosterol was the major sterol component in these samples (>95%). In contrast to the FLU-treated FLU^EVO^ strain, the VOR-treated VOR^EVO^ strain contained a smaller amount of ergosterol (66.26%) and accumulated more lanosterol (13.17%), 14-Me-ergosta-dien-diol (9.22%), and 14-Me-fecosterol (4.43%) and a smaller amount of US2 (1.39%) and US3 (4.91%) (Fig. 5C; Data Set S2). In the case of the POS^EVO^ strain under both the POS-treated and the untreated circumstances, the sterol composition pattern was completely different from that of CLIB 214 or the FLU^EVO^ and VOR^EVO^ strains. Ergosterol was detectable in infinitesimally small amounts in the POS^EVO^ strain in the presence or absence of POS. In the absence of POS, the major sterol component was fecosterol/episterol/ergosta-dienol (92.34%, detected all together). Furthermore, small amounts of another yet unidentified sterol (US1, 3.96%) and ergosta-enol (1.53%) were detected in the POS^EVO^ strain (Fig. 5D; Data Set S2). In the presence of POS, the POS^EVO^ strain mainly produced 14-Me-fecosterol (82.81%) and lanosterol (12.21%) (Fig. 5D; Data Set S2). These data about the POS^EVO^ strain suggest an *ERG3* loss-of-function mutation (Fig. 5A).

10.1128/mSphere.00821-20.2 DATA SET S2 Sterol compositions of the parental and evolved C. parapsilosis strains in the absence and the presence of the corresponding azoles. The data represent the peak areas of the dehydrated molecular ions in the chromatogram. Download Data Set S2, XLSX file, 0.02 MB. Copyright © 2020 Papp et al. 2020 Papp et al. This content is distributed under the terms of the Creative Commons Attribution 4.0 International license.

### Azole microevolution alters the *in vivo* virulence properties of evolved strains in a mouse model.

**FIG 6:**
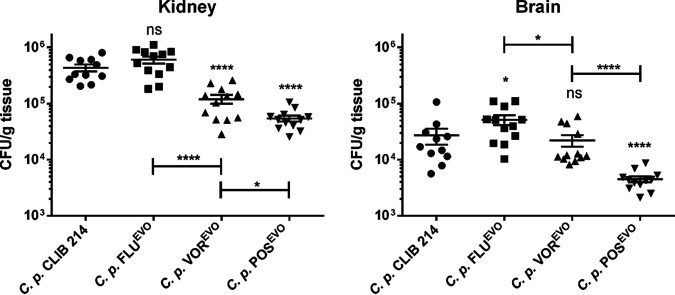
Fungal burden of the brain and kidneys of BALB/c mice at 3 days postinfection. Data are for 12 mice from each group from 3 different experiments. Statistical analyses were performed by Mann-Whitney tests. *, *P* ≤ 0.05; ****, *P* ≤ 0.0001; ns, not significant.

We next investigated the virulence of the triazole-evolved strains in a mouse model of systemic candidiasis. The fungal burdens of the evolved strains in the kidneys and/or brain were altered from those of the parental strain (Fig. 6), but there were no significant changes in the numbers of CFU in the liver and spleen (data not shown). Although adaptation to FLU did not affect kidney colonization, significant decreases in the numbers of CFU recovered after VOR^EVO^ and POS^EVO^ infection compared to the numbers of CFU recovered after infection of this organ with the parental strain were detected. Notably, the number of CFU recovered following infection with the POS^EVO^ strain was significantly lower even than the number of CFU recovered following infection with the VOR^EVO^ strain (Fig. 6). Interestingly, the fungal burden in the brain significantly increased after FLU^EVO^ infection, while no difference was detected in the case of VOR^EVO^ infection, and a significant decrease in the number of CFU was observed after infection with the POS^EVO^ strain relative to the number of CFU seen after infection with the parental strain.

In comparing the virulences of the triazole-evolved strains to each other, we found that the POS^EVO^ strain showed the greatest virulence attenuation in both organs, followed by the VOR^EVO^ strain in the kidneys and the FLU^EVO^ strain, which showed no virulence attenuation. When comparing the findings for the evolved strains to those for the parental strain, we can conclude that POS evolution led to the highest cost of virulence both in the kidneys and in the brain, while VOR evolution resulted in a loss of virulence only in the kidneys and FLU evolution had either no effect or a gain-of-virulence effect in both organs (Fig. 6).

## DISCUSSION

In our previous study, we demonstrated the relationship between acquired echinocandin (caspofungin, anidulafungin, micafungin) resistance and the virulence properties of C. parapsilosis. We found that the development of echinocandin resistance attenuates the virulence of C. parapsilosis *in vivo* independently of the applied echinocandin (10).

Here we aimed to explore the mechanisms of resistance to different triazoles (FLU, VOR, POS) and the consequences of adaptation on stress tolerance and virulence properties. First, we generated three independent triazole-evolved (FLU^EVO^, VOR^EVO^, POS^EVO^) strains by repeatedly passaging C. parapsilosis CLIB 214 cells in the presence of increasing drug concentrations (Fig. 1). Previous studies showed that azole resistance development may also lead to increased MIC values or even resistance to echinocandins (23, 24). Our FLU^EVO^ and VOR^EVO^ strains showed a similar resistance pattern, as these two strains developed resistance to both FLU and VOR; however, the POS MIC did not change and only a 1-fold increase or decrease in the MICs of the other antifungals examined was observed. It is important to note that both the FLU and VOR MIC values appeared to be significantly higher for the VOR^EVO^ strain than for the FLU^EVO^ strain. Despite the increasing number of FLU-VOR cross-resistant isolates (possibly due to shared resistance mechanisms [25]), successful VOR treatments were also reported in experimental and clinical cases of FLU-resistant *Candida* infection. In other cases, FLU treatment was not successful (26, 27). This clinical observation could be an explanation for the different FLU and VOR MIC values for the FLU^EVO^ and VOR^EVO^ strains. The pattern of the MIC values for the POS^EVO^ strain differed significantly from the patterns for the other two azole-evolved strains. VOR is a derivative of FLU, and cross-resistance between these two related azoles is frequent. In contrast, POS is a derivative of itraconazole, and cross-resistance with FLC or VOR is much less frequent (28). Thus, structural relations between the different azoles may explain the pattern of MIC values of the azole-evolved strains. The POS^EVO^ strain possessed the highest MIC value for all tested azoles. In contrast to the findings for the FLU^EVO^ and VOR^EVO^ strains, the POS^EVO^ strain showed increased echinocandin MIC values. The patterns in the resistance of the generated strains anticipated some of the differences in the resistance mechanisms of the different evolved strains. In contrast to the results of the MIC distribution, the POS^EVO^ strain was the most susceptible to all osmotic, cell wall, and plasma membrane stressors. Interestingly, the FLU^EVO^ and the VOR^EVO^ strains were resistant to the cell wall stressor caffeine.

Results from whole-genome sequencing produced insights into potential resistance mechanisms responsible for the different MIC values. According to the sequencing results, the resistance in our FLU^EVO^ and VOR^EVO^ strains possibly developed due to two different SNPs in the *MRR1* gene, responsible for *MDR1* transcription activation (21). However, we did not find any nucleotide changes in *TAC1*, activating constitutive *CDR1* expression (18). It is possible that the different levels of FLU and VOR resistance of the FLU^EVO^ and VOR^EVO^ strains were due to the distinct nature of the amino acid substitutions (A808T and N394Y, respectively); however, there is no evidence in the literature about the effect of alternative SNPs in *MRR1*. The POS^EVO^ strain did not harbor any SNPs in *MRR1* or *TAC1*. Previous studies showed that *MRR1* is not necessarily responsible for increased *MDR1* expression, as the disruption of *MRR1* decreases only FLU MIC values (18). However, the identified *MRR1* mutations in the FLU^EVO^ and VOR^EVO^ strains in this study may suggest otherwise, as these strains showed significantly increased MIC values for FLU and VOR, which was possibly due to elevated efflux pump activity. Interestingly, in the FLU^EVO^ and VOR^EVO^ strains, in addition to the elevated expression of *MDR1*, the expression of CPAR2_405280 and *CDR1* (potential ABC transporters) also increased. These findings and those of Berkow et al. (18) suggest that in C. parapsilosis, expression of the *MDR1* and *CDR* genes is positively regulated via redundant transcription circuits, which are less dependent on gain-of-function SNPs of transcription factors, such as *MRR1* or *TAC1*. Furthermore, the function of a redundant transcriptional system may not be necessarily dependent on the presence of azoles, as we detected elevated expression of *MDR1* and *CDR* genes in untreated samples; however, *MRR1* is clearly a positive regulator of *MDR1* expression. Regarding the efflux activity together with the stress response to caffeine, we hypothesize that caffeine may also be a substrate of efflux pumps, in contrast to the other stressors used in this study. Our results on the MIC distribution together with the resistance mechanisms of the FLU^EVO^ and VOR^EVO^ strains are consistent with the findings in the literature, as POS is not a substrate of MSF or ABC transporters (20, 25, 29). Therefore, during the generation of the POS^EVO^ strain, another path had to be involved in POS resistance development. According to the WGS results, together with the sterol composition data (a lack of sterol compounds downstream of Erg3p and the accumulation of sterol compounds upstream of Erg3p), for the POS^EVO^ strain, we suggest that the mutation in *ERG3* is responsible for a wide range of azole resistance. It is consistent with the findings in the literature that the elevated echinocandin MIC values are connected to *ERG3* loss-of-function mutations, although the exact molecular mechanism regarding how the loss or decrease of C-5 sterol desaturase activity could lead to echinocandin resistance in C. parapsilosis is still unclear (30). It may be hypothesized that the altered sterol content could lead to an elevated stress response, leading to a compensation effect of echinocandins. The first part of this hypothesis was confirmed, as we demonstrated the altered sterol composition of the POS^EVO^ strain. Additionally, the accumulated fecosterol/episterol/ergosta-dienol levels in the POS-untreated POS^EVO^ strain and 14-Me-fecosterol levels in the POS-treated POS^EVO^ samples highlight the loss-of-function characteristic of the *ERG3* mutation in this strain. The sterol composition of the FLU^EVO^ and VOR^EVO^ strains treated with the corresponding azole showed the effectiveness of VOR even in resistant isolates, as the VOR-treated VOR^EVO^ strain produced less ergosterol and accumulated a higher portion of 14-Me-ergosta-dien-diol than the untreated CLIB 214, FLU-untreated FLU^EVO^, and FLU-treated FLU^EVO^ strains. We detected three unidentified sterol components in different strains under different conditions. We plan to elucidate the exact structure of these compounds by nuclear magnetic resonance spectroscopy in collaboration with analytical experts.

Interestingly, the *in vivo* virulence properties of the azole-evolved strains showed a pattern reciprocal to that for the MIC values. For example, the FLU^EVO^ strain was as virulent as the parental CLIB 214 strain, despite having the lowest MIC values of azoles compared to the other azole-evolved strains. Previous studies showed that FLU treatment in Galleria mellonella larvae is ineffective against FLU-resistant C. parapsilosis strains (31). This information, together with our results, indicates that adaptation to FLU does not imply a virulence cost in C. parapsilosis, as in the case of echinocandin adaptation (10). In C. glabrata PDR1 (*CgPDR1*), gain-of-function mutations not only lead to elevated azole MICs but also enhance virulence, indicating that *CgPDR1* possibly regulates uncharacterized virulence factors (32). C. parapsilosis *MRR1* (*CpMRR1*) regulates *MDR1*, but we found elevated expression of *CDR* genes (CPAR2_405280, *CDR1*) in strains bearing SNPs in *MRR1*; therefore, the possibility that *MRR1* regulates the expression of other unidentified genes responsible for virulence in C. parapsilosis cannot be excluded. This hypothesis must be confirmed by further experiments, as the virulence of the VOR^EVO^ strain was attenuated in the kidney but not in the brain of infected mice, despite the shared resistance mechanism with the FLU^EVO^ strain. This can be explained by the distinct *MRR1* mutation, *MDR1* expression, and efflux pump activity or by the uncharacterized changes in the transcriptome of the VOR^EVO^ strain. The virulence of the POS^EVO^ strain was attenuated the most, as the numbers of CFU were the lowest both in the kidneys and in the brain of POS^EVO^-infected mice compared to those of the other strains. The impaired virulence of POS^EVO^ is possibly due to *ERG3* inactivation, as in C. albicans the lack of sterol Δ5,6-desaturase activity leads to decreased virulence (33, 34). However, there is an example in C. albicans in which this resulted in a reduced but not absent expression of *ERG3*, such that resistance to azoles occurred, but no changes in virulence properties were detected (35).

In summary, the POS^EVO^ strain was the least virulent, which was possibly due to its high susceptibility to stressors. Stress tolerance and the virulence pattern were similar in the case of the FLU^EVO^ and VOR^EVO^ strains. This may have been due to the fact that voriconazole is a derivative of fluconazole, as the corresponding strains showed similar phenotypic changes. Based on the genome analysis results, the genes affected by SNPs may be responsible for the differences between the POS^EVO^ and the FLU^EVO^/VOR^EVO^ strains, of which *MRR1* and *ERG3* are annotated in C. parapsilosis. Based on the *Candida* Genome Database, none of the other *CPAR2* genes have an orthologous gene that could be related to antifungal resistance, the stress response, or virulence regulation.

Our results, together with the findings in the literature, highlight the necessity of deeper investigations of antifungal resistance development in C. parapsilosis and other species, as the outcome of different antifungal treatments may depend not only on the effectiveness of administered antifungal drugs but also on the virulence cost effect that could be impacted by resistance mechanisms (36).

## MATERIALS AND METHODS

### Generation of azole-evolved strains.

Triazole-evolved strains were generated as described previously (10, 37), with minor modifications (Fig. 1). To determine the initial concentration of triazoles for the microevolution process, we determined the MIC values of FLU, VOR, and POS for the parental C. parapsilosis CLIB 214 strain (38). For microevolution, the initial concentration of each drug was half of the MIC values determined for the parental strain: 0.5 μg/ml, 0.0156 μg/ml, and 0.0156 μg/ml for FLU, VOR, and POS, respectively.

### Strains and culturing conditions.

The strains utilized in this study are listed in Table 3. Before each experiment, each C. parapsilosis strain was cultured in YPD broth at 30°C for 16 h and then 200-μl aliquots were inoculated into 5 ml fresh YPD liquid medium and incubated at 30°C for 16 h. The parental C. parapsilosis CLIB 214 strain was maintained on YPD, while the FLU^EVO^, VOR^EVO^, and POS^EVO^ strains were maintained on YPD agar plates supplemented with 64 μg/ml, 8 μg/ml, and 8 μg/ml FLU, VOR, and POS, respectively.

**TABLE 3:** C. parapsilosis strains used in this study

Strain	Origin	Reference or source
CLIB 214	Laboratory type strain	38
FLU^ADP^	CLIB 214	This work
VOR^ADP^	CLIB 214	This work
POS^ADP^	CLIB 214	This work
FLU^EVO^	FLU^ADP^	This work
VOR^EVO^	VOR^ADP^	This work
POS^EVO^	POS^ADP^	This work

### Antifungal susceptibility test.

MIC values were determined according to the M27-A3 protocol (39), and MIC values were defined by the M27-S4 supplementary document (40). MICs were measured in RPMI 1640 with MOPS (morpholinepropanesulfonic acid) with l-Gln but without NaHCO_3_ (catalog number 04-525F; Lonza) at 24 h and 48 h. The MIC values for the echinocandins were defined as the lowest concentrations that resulted in at least 50% growth reduction. Amphotericin B (AMP; Sigma-Aldrich), azoles (FLU, VOR, POS, and ITR; Sigma-Aldrich), and echinocandins (CAS [Sigma-Aldrich] and AND and MICA [MedChem Express]) were utilized to test the susceptibilities of the C. parapsilosis strains.

### Abiotic stress tolerance.

The C. parapsilosis CLIB 214, FLU^EVO^, VOR^EVO^, and POS^EVO^ strains were serially diluted, and 10^4^, 10^3^, 10^2^, and 10^1^ cells were transferred in 5 μl to solid YPD plates adjusted to pH 4, pH 5, pH 6, pH 7, or pH 8 (McIlvaine buffer) and to YPD plates without any supplements. For comparing the growth abilities of the triazole-evolved strains to the growth ability of the parental CLIB 214 strain in the presence of osmotic and oxidative stressors as well as cell membrane- and cell wall-perturbing agents, we plated the strains on YPD agar plates complemented with 8% (wt/vol), 10% (wt/vol), or 12% (wt/vol) glycerol, 1 M or 1.5 M NaCl, or 1 M sorbitol (as osmotic stressors); with 0.05 mM CdSO_4_ or 5 mM or 10 mM H_2_O_2_ (as oxidative stressors); with 12.5 mM, 15 mM, or 17.5 mM caffeine; 50-μg/ml, 75-μg/ml, or 100-μg/ml calcofluor white, or 10-μg/ml, 25-μg/ml, 50-μg/ml, or 75-μg/ml Congo red (as cell wall-perturbing agents); or with 0.02% (wt/vol), 0.04% (wt/vol), or 0.06% (wt/vol) SDS (as a membrane-perturbing agent). The plates were incubated at 30°C and 37°C for 48 h. The growth scores of the evolved (EVO) strains were determined and compared to those of the parental strain. All experiments were repeated two times. We defined the defect scores as described previously (10): a score of 1 was a strong defect, in which the growth of the given evolved strain spot, which was three times more concentrated than that of the most diluted CLIB 214 spot in which growth appeared, was reduced (smaller colonies or lower colony numbers); a score of 2 was a medium defect, in which similar numbers of CFU appeared in the given evolved strain spot, which was two times more concentrated than the most diluted CLIB 214 spot in which growth appeared; and a score of 3 was a slight defect, in which the growth in the given evolved strain spot at one time the concentration of the most diluted CLIB 214 spot in which growth appeared was reduced or colonies smaller than the parental strain’s colonies were present.

### *In vivo* infection of mice and determination of fungal burden.

For determining fungal burdens, 10- to 12-week-old female BALB/c mice (catalog number XVI./2015; BRC, Szeged, Hungary) were injected via the lateral tail vein with 2 × 10^7^ yeast cells in 100 μl phosphate-buffered saline (PBS) (*n* ≥ 10 mice per C. parapsilosis strain). At 3 days postinfection, the animals were euthanized and the liver, spleen, kidneys, and brain were collected, weighed, and homogenized with an Ultra-Turrax T25 homogenizer (Sigma). Organ homogenates were plated onto YPD agar plates supplemented with 1% penicillin-streptomycin, and the numbers of CFU were counted after 48 h of incubation at 30°C.

### Ethics statement.

Animal experiments were performed according to Hungarian national animal ethics guidelines (guideline 1998, XXVIII; 40/2013) and European animal ethics guidelines (guideline 2010/63/EU). The procedures were licensed by the Animal Experimentation and Ethics Committee of the Biological Research Centre of the Hungarian Academy of Sciences and the Hungarian National Animal Experimentation and Ethics Board (clearance number XVI./03521/2011), with the University of Szeged granting permission XII./00455/2011 and XVI./3646/2016 to work with mice.

### Genome sequencing.

Libraries for whole-genome DNA sequencing were prepared with a Nextera XT DNA library preparation kit (Illumina) with Nextera XT index kit adapters following the manufacturer’s protocol for sequencing runs of ≥2 × 250 cycles. The sequencing libraries were validated and quantitated with an Agilent 2100 Bioanalyzer capillary electrophoresis instrument using an Agilent DNA 1000 kit. After pooling and denaturing, the libraries were sequenced with an Illumina MiSeq sequencer using a MiSeq reagent kit (version 3-600). Th paired-end read length was 2 × 300 bp, and the final average per base sequencing depth ranged from 46 to 130 times.

### Genome analysis of C. parapsilosis strains.

Paired-end fastq reads files were first trimmed using the Trimmomatic tool (version 0.36) (41). The parameters employed were as follows: we removed leading and trailing nucleotides with a quality below 10 (the LEADING and TRAILING parameters, respectively), we used 4-nucleotide sliding windows and cut when the average quality per nucleotide in a window was below 15 (the SLIDINGWINDOW parameter), and we dropped any reads that were less than 40 nucleotides after this trimming (the MINLEN parameter). Then, we mapped the trimmed reads with the bwa-mem tool from BWA (version 0.7.12-r1039) software (42). The reference genome against which the reads were mapped was the CDC317 strain fasta file obtained in April 2018 from the *Candida* Genome Database (43). We generated BAM files from this output using the SortSam and MarkDuplicates commands from the Picard (version 2.15.0) set of tools (http://broadinstitute.github.io/picard/). Finally, we called variants from these reads using the Freebayes (version 1.1.0-9-g09d4ecf) program (44) to jointly genotype all the strains involved. We filtered the SNPs using the vcffilter tool from the vcflib library (E. Garrison, https://github.com/vcflib/vcflib). We removed any SNPs for which the mean mapping quality (MQM) value was below 30, the QUAL (quality) value was below 20 (indicating a probability of a false variant call greater than 0.01), and/or the read depth (DP) was below 30.

### Analysis of calcein efflux of C. parapsilosis strains by flow cytometry.

C. parapsilosis CLIB 214 and the evolved strains were washed with Hanks balanced salt solution (HBSS; Lonza) three times, and then 10^9^ cells of each strain were coincubated with 20 μg/ml calcein-AM (Merck) in 300 μl HBSS for 90 min with gentle shaking at 30°C. Next, the cells were washed with HBSS three times and incubated in HBSS supplemented with 1% glucose without calcein-AM for 90 min.

After the second 90-min incubation, the cells were washed three times with PBS without glucose and were resuspended in 200 μl PBS. Unstained cells were used as negative controls. The fluorescence intensity of 10^4^ cells of each strain was examined using an Amnis Flow Sight imaging flow cytometer with a 488-nm laser (Amnis, Merck Millipore, Billerica, MA, USA). Data analysis was performed using image data exploration and analysis software (IDEAS; Amnis, Merck Millipore, Billerica, MA, USA).

### RNA extraction and qRT-PCR analysis of *MDR1* expression.

The C. parapsilosis CLIB 214, FLU^EVO^, VOR^EVO^, and POS^EVO^ strains were cultured for 16 h at 30°C. Following centrifugation, 10^7^ cells were inoculated into 1 ml liquid YPD medium with 1% penicillin-streptomycin and incubated at 30°C for 3 h. Next, the cells were collected and washed three times with PBS and then stored in liquid nitrogen for later RNA extractions.

RNA extraction was performed using a RiboPure RNA purification kit, yeast (catalog number AM1926; Ambion), according to the manufacturer’s recommendations. An additional DNase treatment step was applied using an RNase-free DNase set (catalog number 79254; Qiagen). DNA contamination in purified RNA samples was checked by quantitative real-time PCR (qRT-PCR). cDNA synthesis was performed from RNA samples containing 500 ng RNA, using a RevertAid first-strand cDNA synthesis kit (catalog number K1622; Thermo Scientific). The qRT-PCR experiments were performed in triplicate, using *MDR1*-, CPAR2_405280-, or CPAR2_405290-specific and *TUB4*-specific primers (*TUB4* was the housekeeping gene that served as an internal control) (Table 4) and a Maxima SYBR green/fluorescein quantitative PCR master mix (catalog number K0243; Thermo Scientific) in a CFX96 real-time system on a C1000 thermal cycler (Bio-Rad). Expression is shown as the fold change, established by ΔΔ*C_T_* threshold cycle (*C_T_*) analysis.

**TABLE 4:** Primers used in this study

Primer	Sequence
CpTUB4_ReTi_F	5′-GAACACTTATGCCGAGGACAAC-3′
CpTUB4_ReTi_R	5′-CTCTCACCACTGACTCCTTGC-3′
Cp405280_ReTi_F	5′-AGAGTATATGCAACCATACATGAGC-3′
Cp405280_ReTi_R	5′-GATTGCAAGTACTGATTGGTACTGC-3′
Cp405290_ReTi_F	5′-TCGGTTAATGCAAGGTACAGCG-3′
Cp405290_ReTi_R	5′-CCGTCAACACAATGTTGATGGC-3′
CpMDR1_ReTi_F	5′-TTATATGGGCGCATCATTCAAGC-3′
CpMDR1_ReTi_R	5′-GGAAACACCGAGGCAATAGTCG-3′

### Determining the sterol composition by LC-HRMS.

**(i) Sample preparation.** Cell suspensions were adjusted to 10^7^ cells/ml. Azole-exposed samples were treated with 32-μg/ml FLU, 8-μg/ml VOR, or 8-μg/ml POS. All samples were incubated at 30°C in liquid YPD medium for 24 h. Sample preparation was performed as described by Varga et al., with some modifications (45). The freeze-dried samples (10 mg) were saponified with 2 ml of 10% KOH in methanol at 80°C for 90 min. Water (500 μl) and *n*-hexane (1 ml) were added to the cooled samples, and the mixtures were vortexed for 0.5 min. After separation of the phases, the *n*-hexane phase was transferred to a 2-ml vial and evaporated to dryness under N_2_. The extraction with *n*-hexane was repeated twice. The dried extracts were dissolved in 300 μl of methanol and filtered through a 0.2-μm-pore-size polytetrafluoroethylene membrane filter.

**(ii) LC-HRMS.** Chromatographic analysis of the samples was performed using a DionexUltimate 3000 ultra-high-performance liquid chromatography system equipped with a membrane degasser, a binary pump, a standard autosampler, a thermostated column compartment, and a variable-wavelength detector. The sterols were separated on a Gemini-NX C_18_ column (particle size, 3 μm; 150 by 2 mm; Phenomenex) equipped with a Gemini-NX C_18_ guard column (particle size, 5 μm; 4 by 2 mm) with a gradient of methanol-water (9/1, vol/vol) as mobile phase A and methanol as mobile phase B at 40°C. The gradient elution was performed as follows: 0.0 min, 0% mobile phase B; 1.0 min, 0% mobile phase B; 5.0 min, 100% mobile phase B; 10.0 min, 100% mobile phase B; 10.5 min, 0% mobile phase B; 15.0 min, 0% mobile phase B. The flow rate was set to 0.4 ml/min. The injection volume was 5 μl.

Mass analyses were carried out with a Q Exactive Plus hybrid quadrupole-Orbitrap mass spectrometer equipped with an atmospheric pressure chemical ionization (APCI) ion source operating in the positive ion mode. The temperature of the ion transfer capillary, the spray voltage, the sheath gas flow rate, and the auxiliary gas flow rate were set to 350°C, 4.5 kV, 40 arbitrary units, and 10 arbitrary units, respectively. All data were acquired using the full-scan/ddMS2 (full-scan/data-dependent tandem mass spectrometry) mode. All full-scan data were acquired over an *m/z* range of 300 to 500 at a resolution of 70,000 FWHM (full width at half maximum) with a 1.0 × 10^6^ automatic gain control (AGC) target and 100 ms of maximum ion injection time. The acquired data were processed using the Xcalibur (version 2.2.1) and Trace Finder (version 3.3) programs (Thermo Fisher Scientific). The identification of the compounds was based on high-resolution accurate mass analysis when reference standards were not available.

### Statistical analysis.

Statistical analysis was performed and statistical significance was determined using GraphPad Prism (version 6.01) software. Nonparametric Mann-Whitney tests were applied. Results are presented as the mean and standard deviation. Differences between the groups examined were considered statistically significant when the *P* value was <0.05.

### Accession number(s).

Raw sequencing data for the wild-type CLIB 214 strain are available under BioProject accession number PRJNA493002 with SRA accession number SRR7898457. Sequence data for the evolved strains are available under BioProject accession number PRJNA649004, and the SRA accession numbers for the strains are as follows: SRR12333224 (FLU^EVO^), SRR12333223 (VOR^EVO^), and SRR12333222 (POS^EVO^).
